# Supplementary material for: UDP-glucosyltransferase OsUGT75A promotes submergence tolerance during rice seed germination
Source: Nat Commun. 2023 Apr 21;14:2296. doi: 10.1038/s41467-023-38085-5 (PMC10121563; doi:10.1038/s41467-023-38085-5)
Supplement: Supplementary file 1 — Supplementary Information [file 41467_2023_38085_MOESM1_ESM.pdf]

UDP-glucosyltransferase OsUGT75A promotes submergence tolerance during rice seed germination

He *et al.*

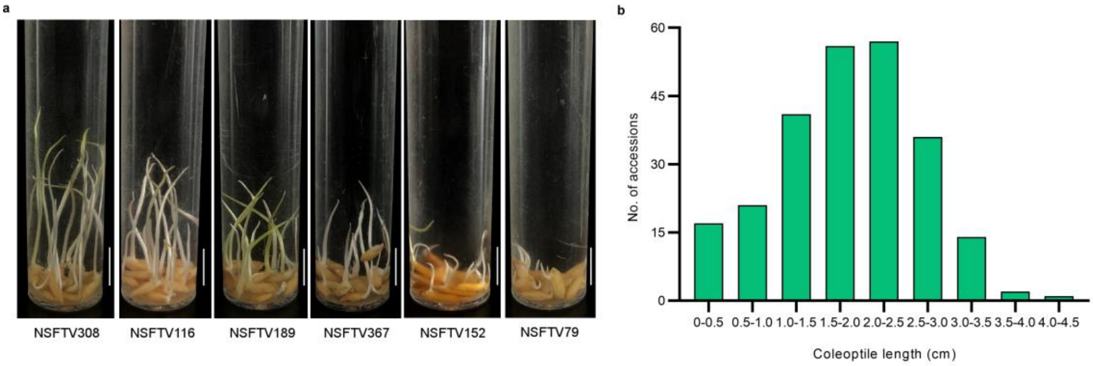

Supplementary Fig. 1. Natural variation of coleoptile length under submergence in rice. a Representative images of coleoptile length among accessions under submergence (8 cm depth of water) for 7 days. Scale bars represent 10 mm. **b** Distribution of coleoptile length among accessions. Information of 245 rice accessions used for GWAS are provided in Supplementary Data 1.

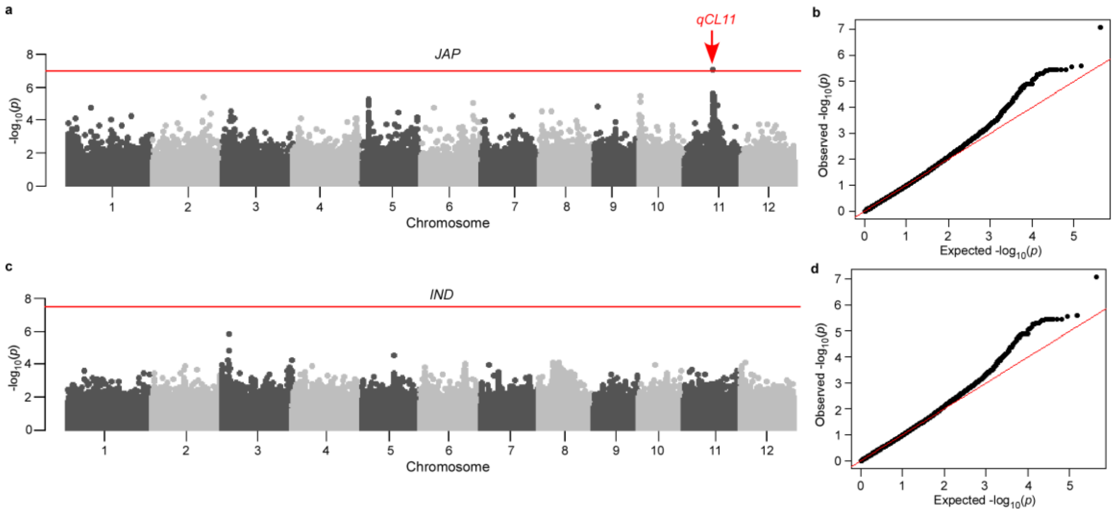

Supplementary Fig. 2. Identification of the associated loci for coleoptile length under submergence in rice. Manhattan plots for the **a** *japonica* and **c** *indica* population. **b**, **d** Quantile-Quantile (QQ) Plots. The red arrow indicates the identified locus. In **a**, **b** no data adjustments were made for GWAS with a threshold of 4.43×10^{-8} (0.01 significance level). In **c**, **d** no data adjustments were made for GWAS with a threshold of 2.85×10^{-8} (0.01 significance level).

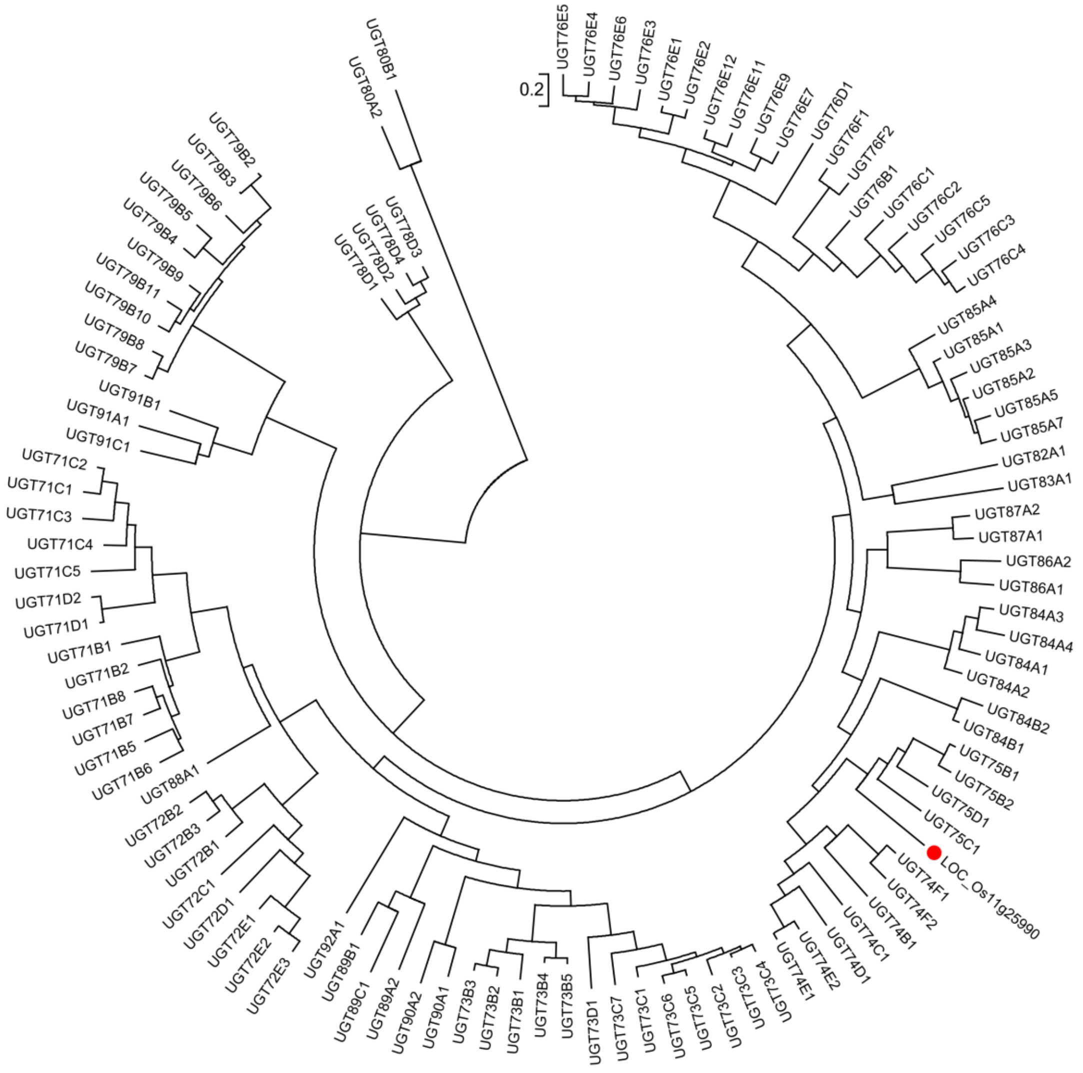

Supplementary Fig. 3. Phylogenetic relationships of rice LOC_Os11g25990 and *Arabidopsis* UDP-glucosyltransferases gene family. The tree was generated using the neighbor-joining method with bootstrap sampling (1000 replicates) using MEGA software version 6.06.

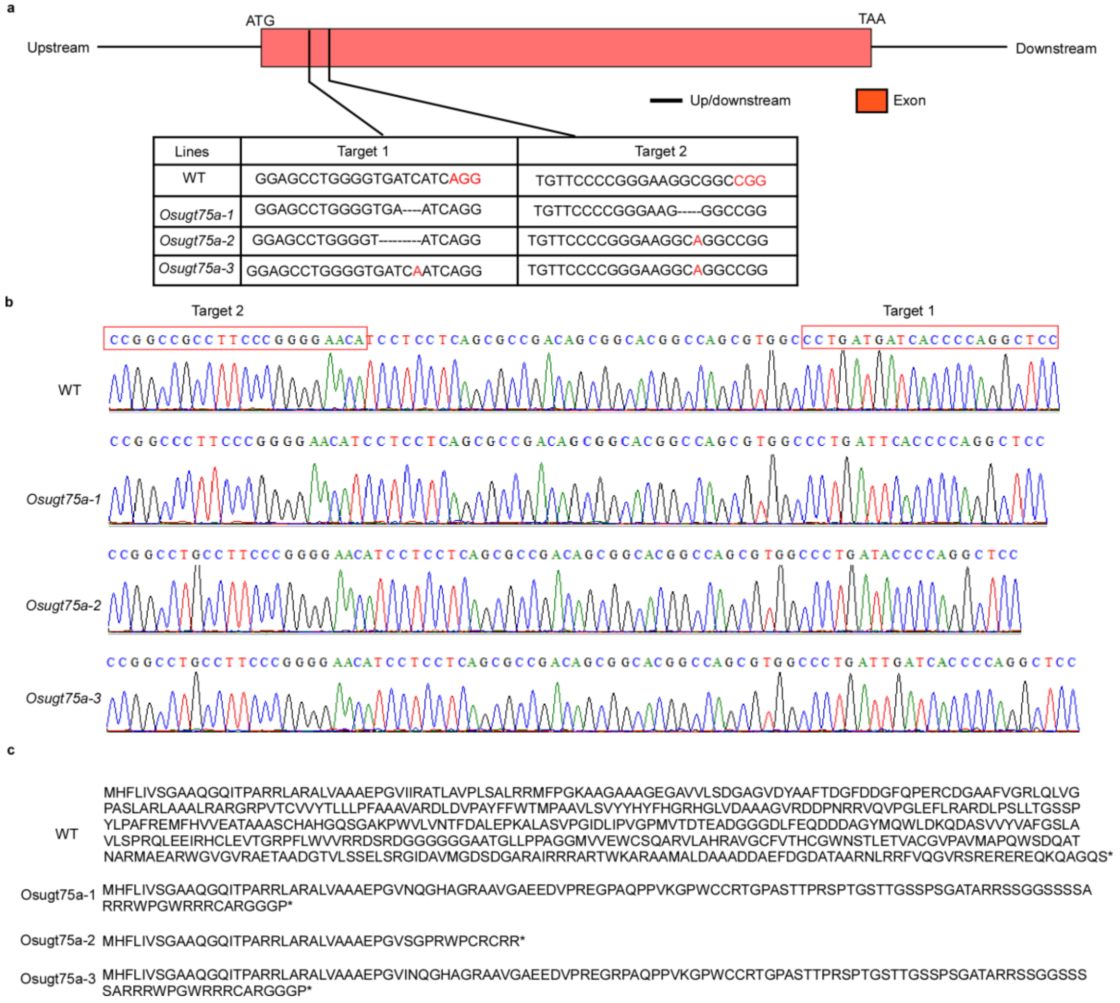

Supplementary Fig. 4. The mutants were generated using the CRISPR/Cas9 system in rice. a The CRISPR targets of *OsUGT75A*. Comparison of **b** nucleotides and **c** amino acid sequences of OsUGT75A between wild-type (WT) Nipponbare and *Osugt75a* mutants in rice.

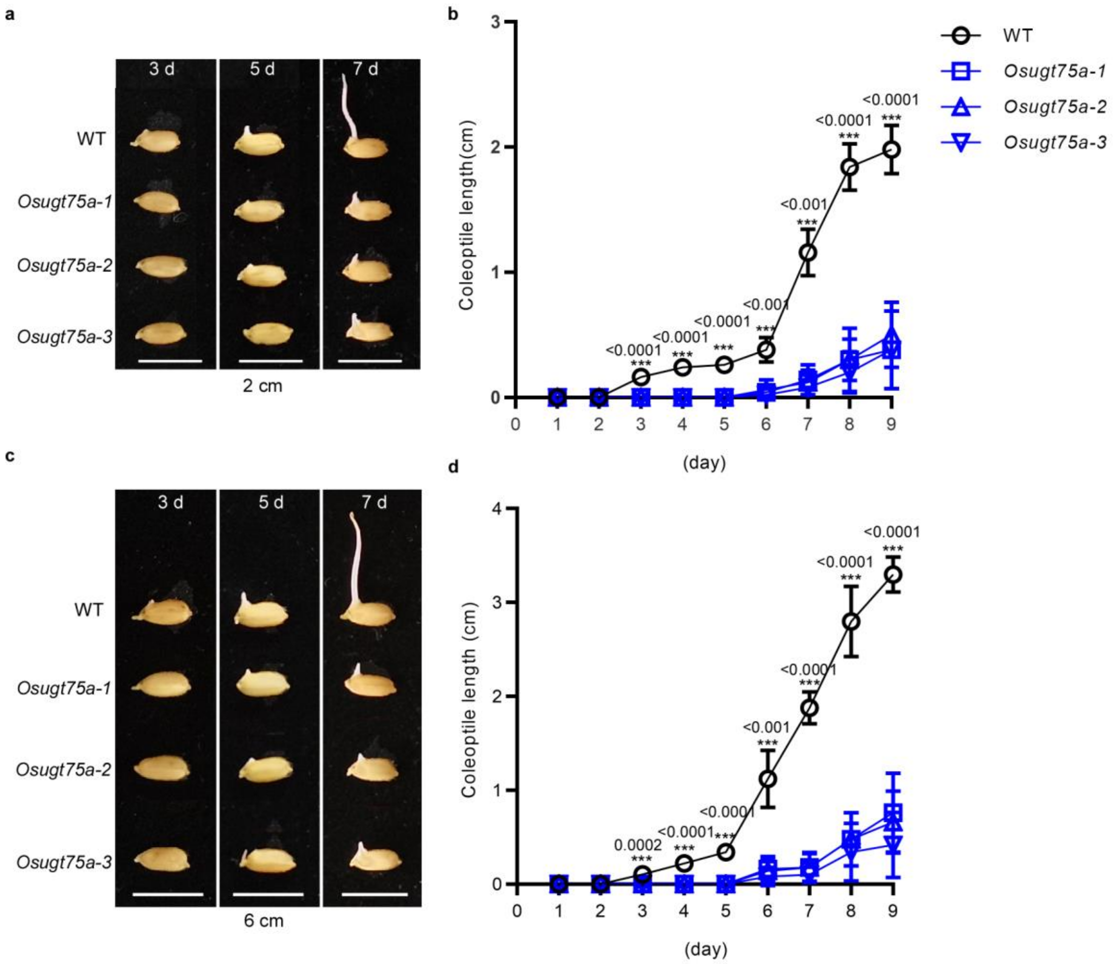

Supplementary Fig. 5. Comparison of coleoptile length between Nipponbare wild-type (WT) and *Osugt75a* mutants under various submergence conditions. a, **c** Representative images of coleoptile length and **b**, **d** dynamic changes of coleoptile length among WT and *Osugt75a* mutants under 2 and 6 cm depth of water for 9 days. Scale bars represent 10 mm. In **b**, **d** data were presented as mean ± SD, $n = 5$; significant differences were determined by two-tailed Student's t-tests (***$P < 0.001$). Source data are provided as a Source Data file.

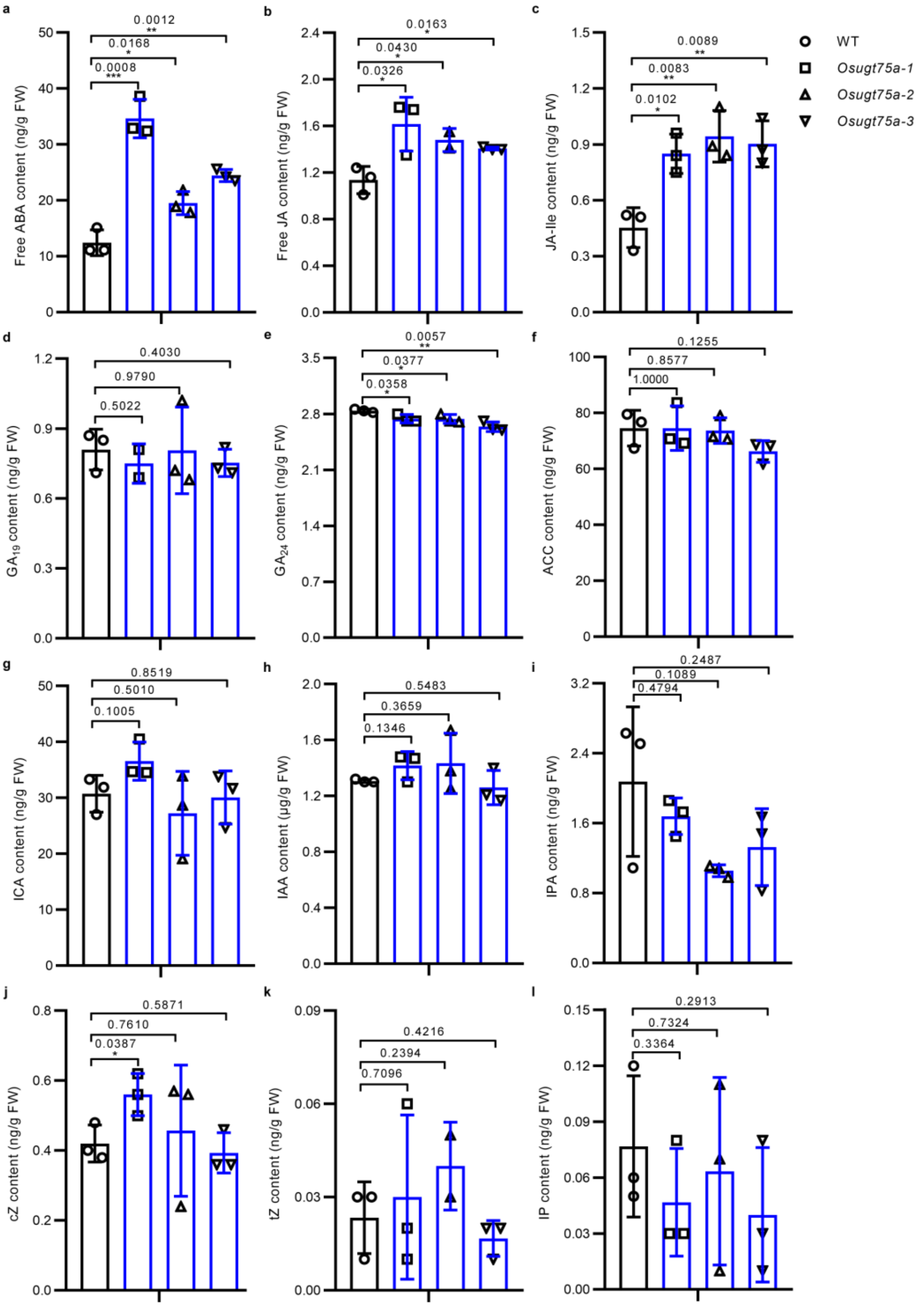

Supplementary Fig. 6. Comparison of the content of free abscisic acid (ABA), jasmonic acid (JA), gibberellins, ethylene, auxins (ICA, IAA, and IPA), and cytokinins (cZ, tZ, and IP) in germinating seeds after 3 days of submergence (8 cm depth of water) in Nipponbare wild-type (WT) and *Osugt75a* mutants. a ABA; **b** JA; **c** JA-Ile; **d** GA_{19}; **e** GA_{24}; **f** Ethylene precursor 1-aminocyclopropane-1-carboxylic acid (ACC); **g** ICA; **h** IAA; **i** IPA; **j** cZ; **k** tZ; **l** IP. In **a**, **b**, **c**, **d**, **e**, **f**, **g**, **h**, **i**, **j**, **k**, **l** data were presented as mean ± SD, n = 3 biologically independent samples. In **a**, **b**, **c**, **e** significant differences were determined by two-tailed Student's t-tests (*P< 0.05, **P < 0.01, ***P < 0.001). Source data are provided as a Source Data file.

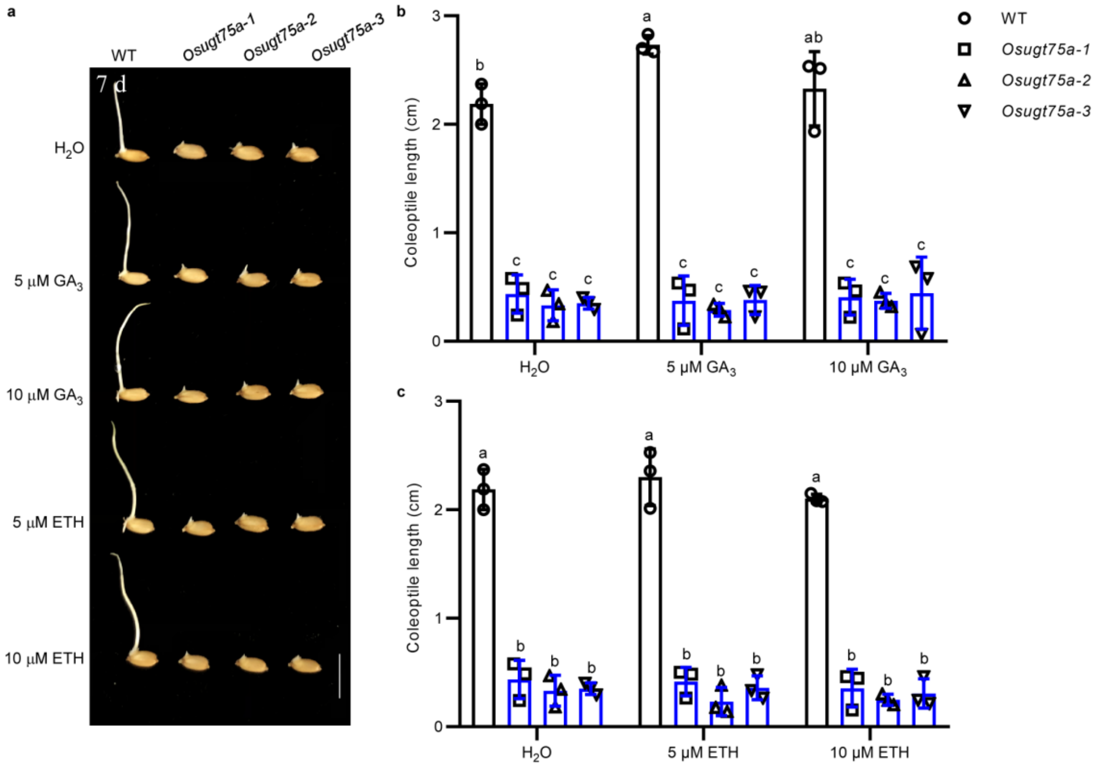

Supplementary Fig. 7. Comparison of the effects of gibberellin (GA_3) and ethephon (ETH) treatments on coleoptile length among Nipponbare wild-type (WT) and *Osugt75a* mutants under submergence. a Representative images of coleoptile length of WT and *Osugt75a* mutants under GA_3 and ETH treatments after 7 days submergence (8 cm depth of water). Scale bar represents 10 mm. **b**, **c** Comparison of coleoptile length among normal, GA_3 and ETH treatments in WT and *Osugt75a* mutants after 7 days submergence. In **b**, **c** data were presented as mean ± SD, $n = 3$ independent experiments; different letters indicate significant differences ($P = 0.05$, one-way ANOVA). Source data are provided as a Source Data file.

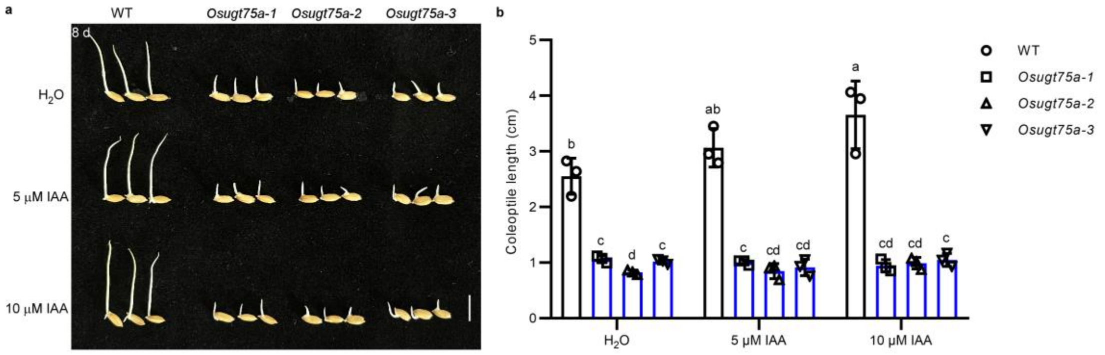

Supplementary Fig. 8. Comparison of the effects of auxin (IAA) treatments on coleoptile length among Nipponbare wild-type (WT) and *Osugt75a* mutants under submergence. a Representative images of coleoptile length of WT and *Osugt75a* mutants under IAA treatments after 8 days submergence (8 cm depth of water). Scale bar represents 10 mm. **b** Comparison of coleoptile length among normal and IAA treatments in WT and *Osugt75a* mutants after 8 days submergence. In **b** data were presented as mean ± SD, $n = 3$ independent experiments; different letters indicate significant differences ($P = 0.05$, one-way ANOVA). Source data are provided as a Source Data file.

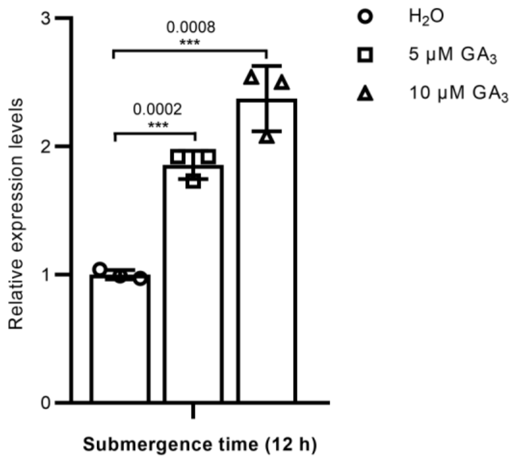

Supplementary Fig. 9. Expression of *OsUGT75A* regulated by GA_3 treatment in *japonica* Nipponbare under submergence (8 cm depth of water) during seed germination. The relative expression levels of *OsUGT75A* were determined by qRT-PCR analysis. *OsActin* gene was used as an internal control. Data were presented as mean ± SD, $n = 3$ biologically independent samples; significant differences were determined by two-tailed Student's t-tests (***$P < 0.001$). Source data are provided as a Source Data file.

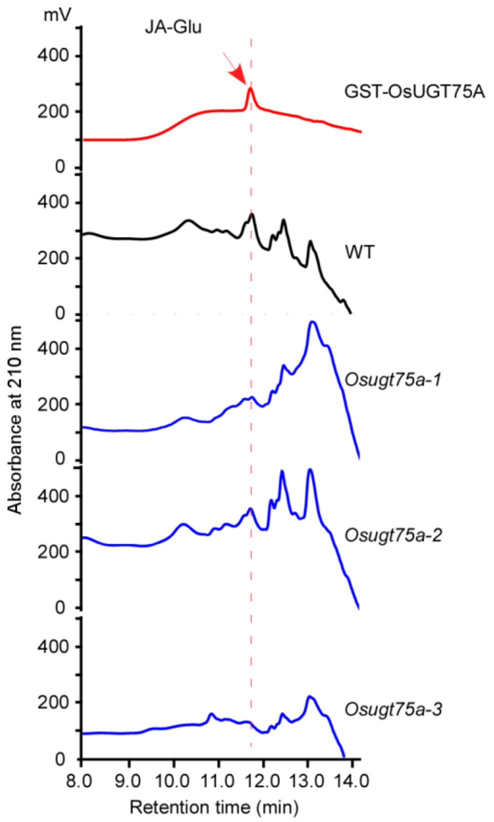

Supplementary Fig. 10. Identification of JA-Glu in coleoptiles in Nipponbare wild-type (WT) and *Osugt75a* mutants under submergence (8 cm depth of water) by targeted HPLC assay. Arrow indicates the product JA-Glu.

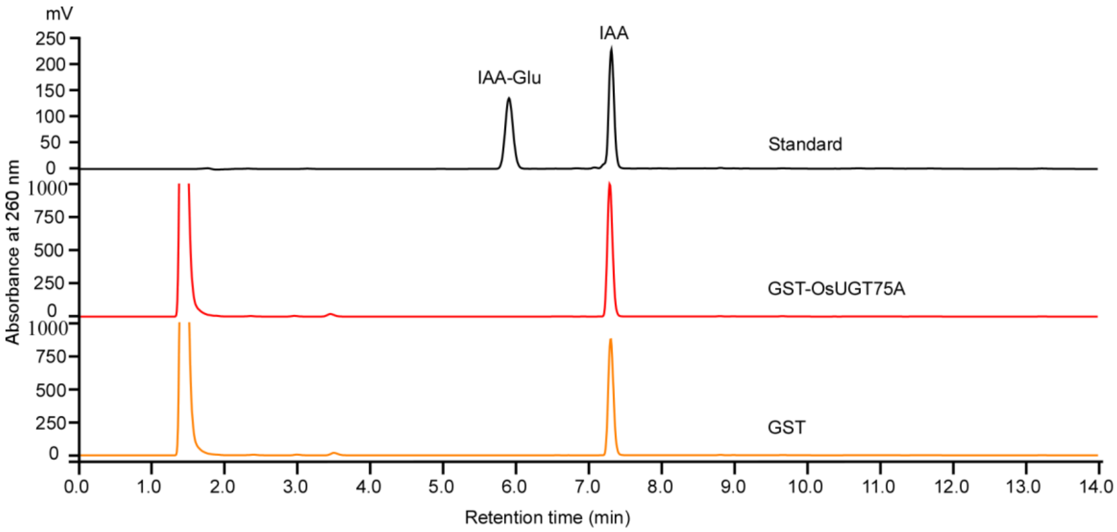

Supplementary Fig. 11. HPLC chromatograms of the reaction of OsUGT75A with UDP-glucose and indole-3-acetic acid (IAA) chromatograms respectively show the absorption at 260 nm.

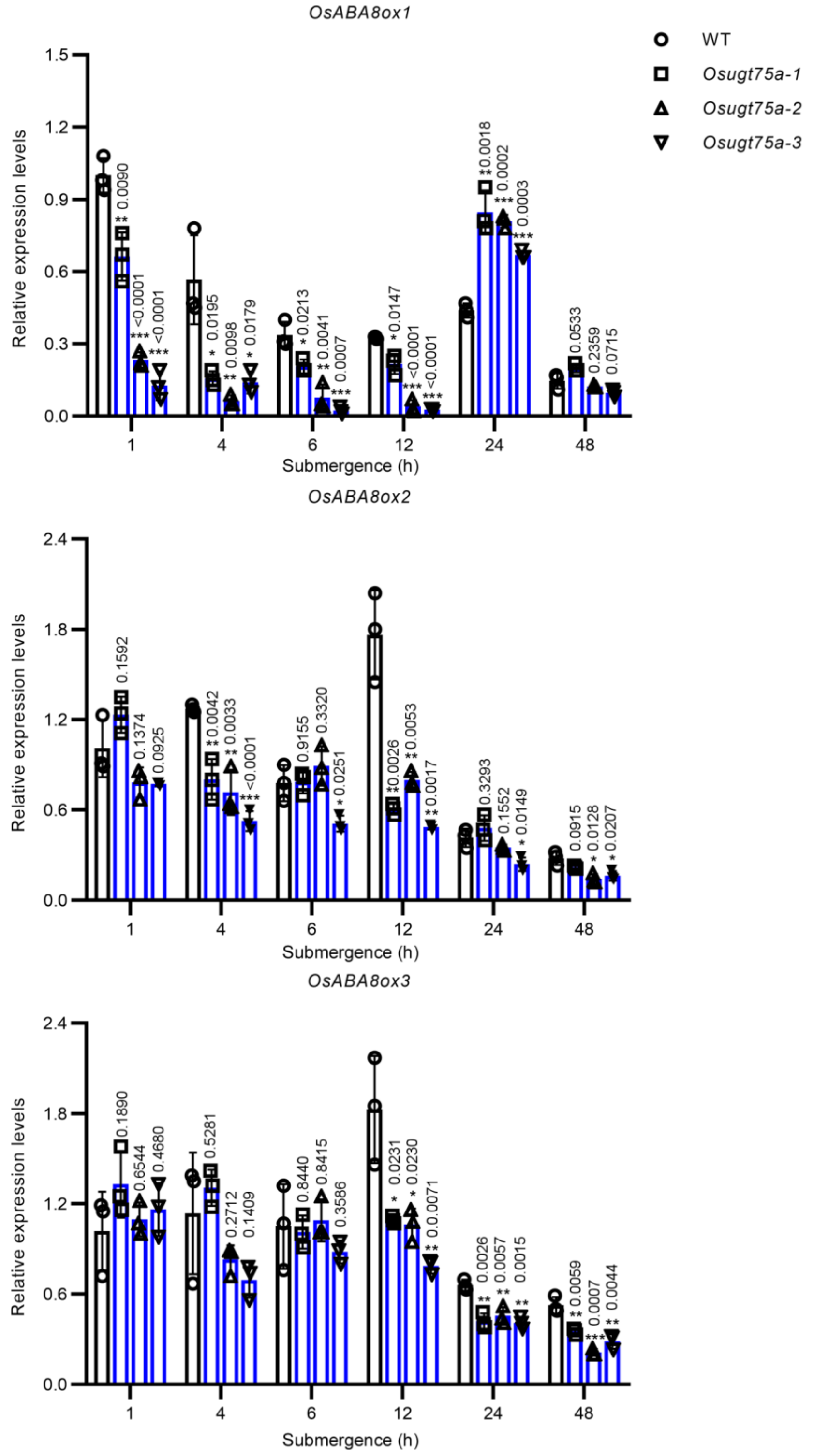

Supplementary Fig. 12. The expression of ABA 8'-hydroxylase (*OsABA8ox*) genes among Nipponbare wild-type (WT) and *Osugt75a* mutants under submergence (8 cm depth of water) determined by qRT-PCR. Expression is shown relative to that in the WT, the value of which was set to 1, with the *OsActin* gene being used as an internal control. Data were presented as mean ± SD, $n = 3$ biologically independent samples; significant differences were determined by two-tailed Student's *t*-tests (*$P< 0.05$, **$P < 0.01$, ***$P < 0.001$). Source data are provided as a Source Data file.

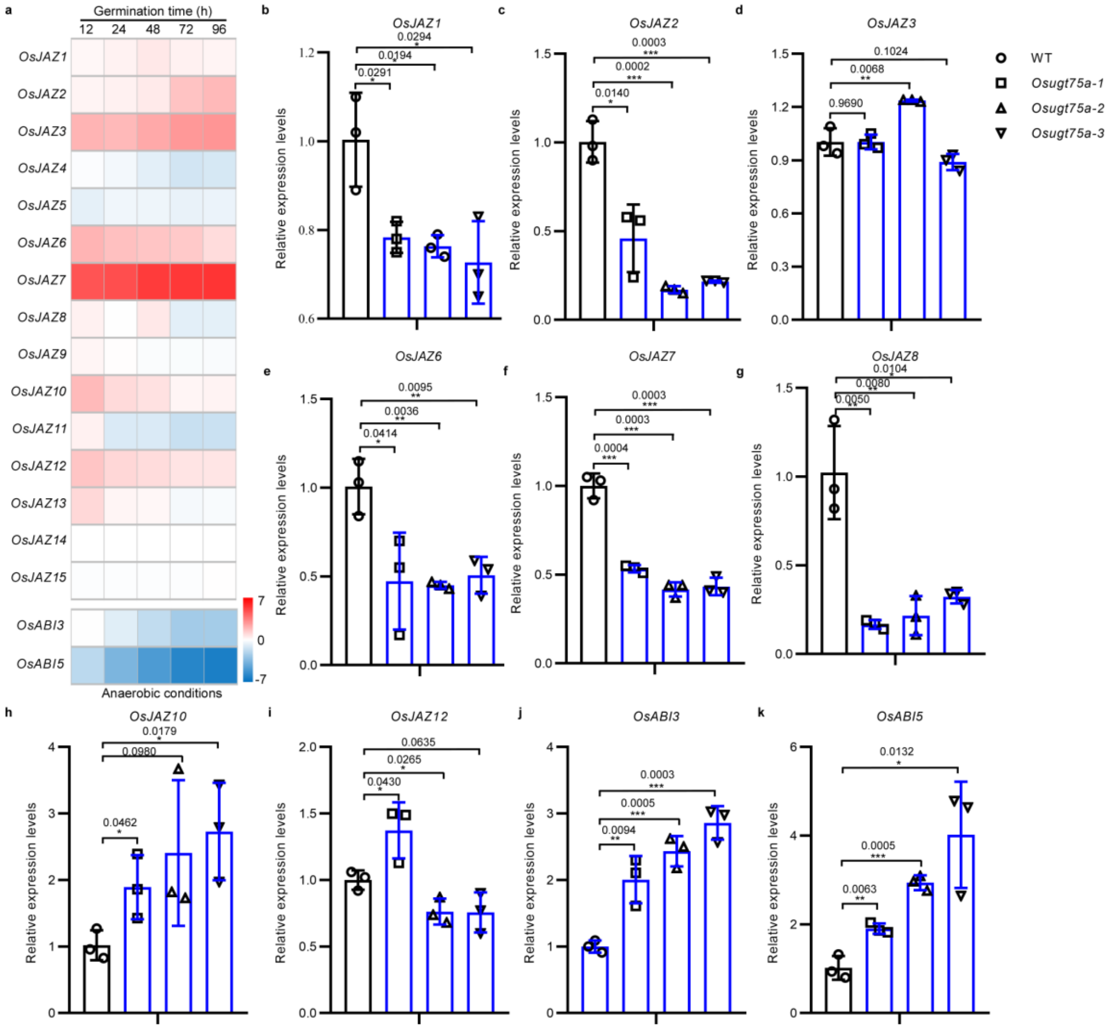

Supplementary Fig. 13. The expression pattern of *OsJAZs* and *OsABIs* among Nipponbare wild-type (WT) and *Osugt75a* mutants under submergence. a The expression of *OsJAZ* and *OsABI* genes in the germinating seeds of *japonica* Nipponbare under submergence was conducted by using the publicly available data (http://www.genevestigator.com). Red, up-regulation; Blue, down-regulation. Values represent the $\log_2$ fold changes of genes. **b**, **c**, **d**, **e**, **f**, **g**, **h**, **i**, **j**, **k** Comparison of the relative expression levels of *OsJAZ* and *OsABI* genes in WT and *Osugt75a* mutants under submergence (8 cm depth of water) determined by qRT-PCR. Expression is shown relative to that in the WT, the value of which was set to 1, with the *OsActin* gene being used as an internal control. In **b**, **c**, **d**, **e**, **f**, **g**, **h**, **i**, **j**, **k** data were presented as mean ± SD, n = 3 biologically independent samples; significant differences were determined by two-tailed Student's t-tests (*P< 0.05, **P < 0.01, ***P < 0.001). Source data are provided as a Source Data file.

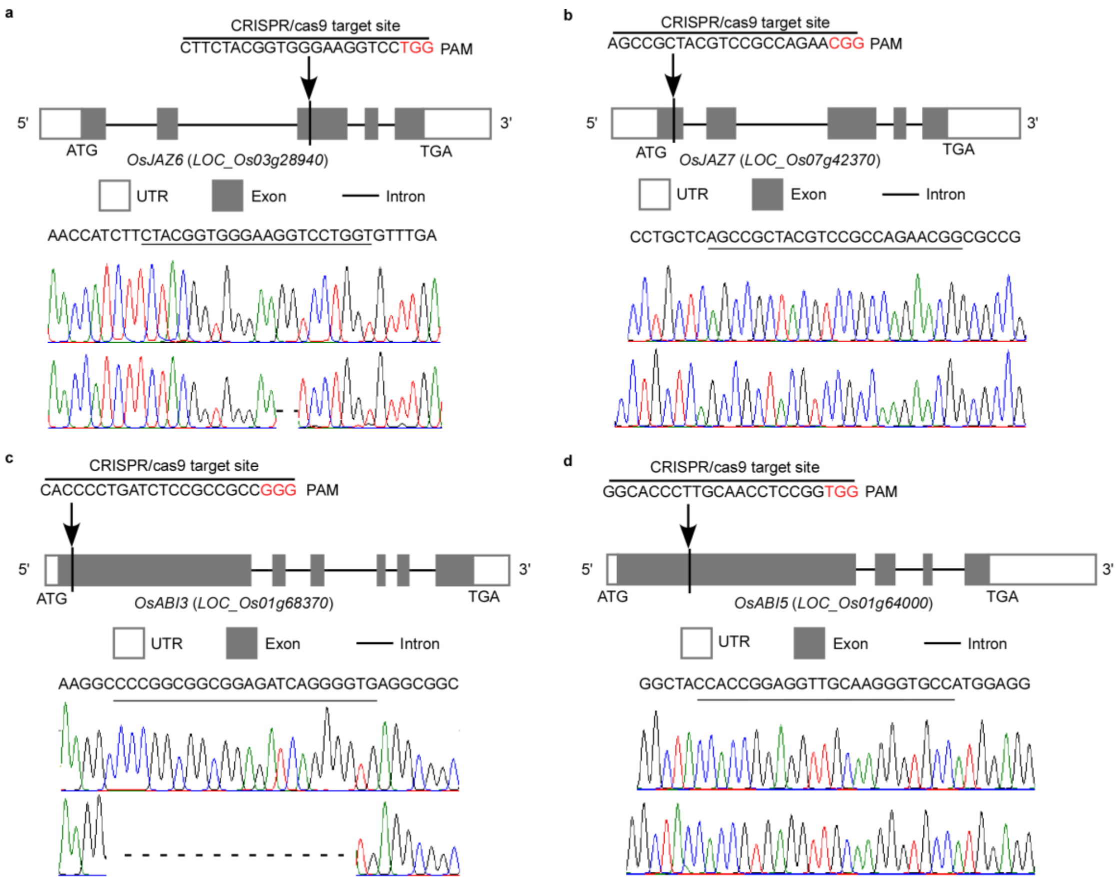

Supplementary Fig. 14. The mutants of *OsJAZ6/7* and *OsABI3/5* were generated using the CRISPR/Cas9 system in rice. a The CRISPR targets of *OsJAZ6*. **b** The CRISPR targets of *OsJAZ7*. **c** The CRISPR targets of *OsABI3*. **d** The CRISPR targets of *OsABI5*.

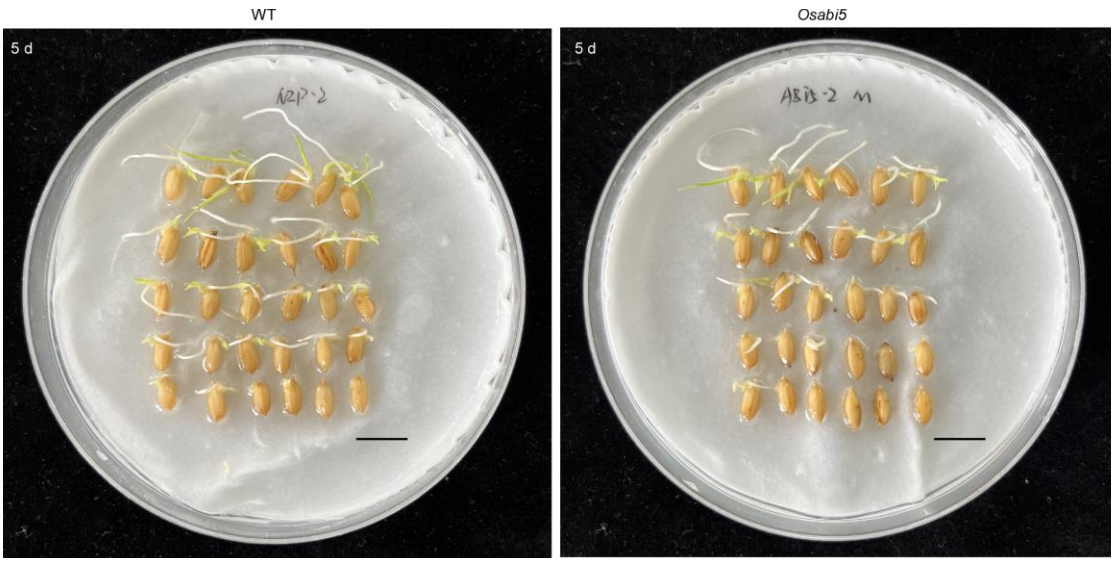

Supplementary Fig. 15. Comparison of seed vigor between Nipponbare wild-type (WT) and *Osabi5* mutant under normal conditions. Scale bars represent 10 mm.

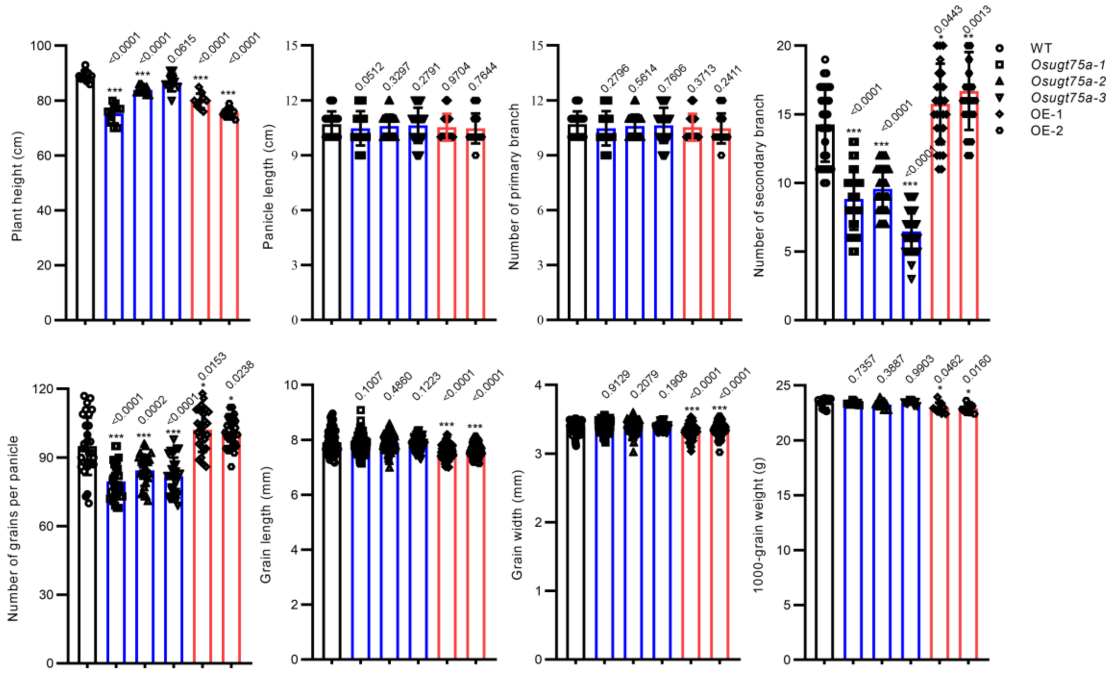

Supplementary Fig. 16. Comparison of agronomic traits among Nipponbare wild-type (WT), *Osugt75a* mutants and overexpression lines. Data were presented as mean ± SD, *n* = 10 for plant height and 1000-grain weight, *n* = 30 for panicle length, number of primary branch, number of secondary branch, and number of grains per panicle, *n* = 99/100/100/88/100/100 grains for grain length and grain width; significant differences were determined by two-tailed Student's *t*-tests (**P*< 0.05, ***P* < 0.01, ****P* < 0.001). Source data are provided as a Source Data file.

Supplemental Table 1. Candidate genes predicted for *qCL11* in rice.

Locus name	Gene product name
LOC_Os11g25890	Hypothetical protein
LOC_Os11g25900	HAT dimerisation domain containing protein
LOC_Os11g25910	Hypothetical protein
LOC_Os11g25920	Hypothetical protein
LOC_Os11g25970	Hypothetical protein
LOC_Os11g25980	Phospholipid-translocating P-type ATPase
LOC_Os11g25990	UDP-glucoronosyl and UDP-glucosyl transferase family protein
LOC_Os11g26020	Hypothetical protein
LOC_Os11g26030	GTP-binding proteins
