# Supplementary material for: UDP-glucosyltransferase OsUGT75A promotes submergence tolerance during rice seed germination
Source: Nat Commun. 2023 Apr 21;14:2296. doi: 10.1038/s41467-023-38085-5 (PMC10121563; doi:10.1038/s41467-023-38085-5)
Supplement: Supplementary file 3 — Description of Additional Supplementary Files [file 41467_2023_38085_MOESM3_ESM.pdf]

Description of Additional Supplementary Files

File Name: Supplementary Data 1
Description: Information of 245 rice accessions used for genome-wide association studies

File Name: Supplementary Data 2
Description: Information of SNPs used for haplotype analysis

File Name: Supplementary Data 3
Description: Information of UDP-glucosyltransferase genes in Arabidopsis

File Name: Supplementary Data 4
Description: Comparison of SNP diversity of OsUGT75A in the currently cultivated *japonica* and *indica* in China

File Name: Supplementary Data 5
Description: The primer pairs used in this study
