# Supplementary material for: UDP-glucosyltransferase OsUGT75A promotes submergence tolerance during rice seed germination
Source: Nat Commun. 2023 Apr 21;14:2296. doi: 10.1038/s41467-023-38085-5 (PMC10121563; doi:10.1038/s41467-023-38085-5)
Supplement: Supplementary file 9 — Reporting Summary [file 41467_2023_38085_MOESM9_ESM.pdf]

nature portfolio

Corresponding author(s): Zhoufei Wang

Last updated by author(s): Apr 12, 2023

Reporting Summary

Nature Portfolio wishes to improve the reproducibility of the work that we publish. This form provides structure for consistency and transparency in reporting. For further information on Nature Portfolio policies, see our Editorial Policies and the Editorial Policy Checklist.

Statistics

For all statistical analyses, confirm that the following items are present in the figure legend, table legend, main text, or Methods section.

n/a	Confirmed	
☐	☒	The exact sample size (*n*) for each experimental group/condition, given as a discrete number and unit of measurement
☐	☒	A statement on whether measurements were taken from distinct samples or whether the same sample was measured repeatedly
☐	☒	The statistical test(s) used AND whether they are one- or two-sided<br>*Only common tests should be described solely by name; describe more complex techniques in the Methods section.*
☒	☐	A description of all covariates tested
☒	☐	A description of any assumptions or corrections, such as tests of normality and adjustment for multiple comparisons
☐	☒	A full description of the statistical parameters including central tendency (e.g. means) or other basic estimates (e.g. regression coefficient) AND variation (e.g. standard deviation) or associated estimates of uncertainty (e.g. confidence intervals)
☐	☒	For null hypothesis testing, the test statistic (e.g. *F*, *t*, *r*) with confidence intervals, effect sizes, degrees of freedom and *P* value noted<br>*Give P values as exact values whenever suitable.*
☒	☐	For Bayesian analysis, information on the choice of priors and Markov chain Monte Carlo settings
☒	☐	For hierarchical and complex designs, identification of the appropriate level for tests and full reporting of outcomes
☒	☐	Estimates of effect sizes (e.g. Cohen's *d*, Pearson's *r*), indicating how they were calculated

Our web collection on statistics for biologists contains articles on many of the points above.

Software and code

Policy information about availability of computer code

Data collection	CFX ManagerTM Software Version 3.1 (qRT-PCR data); GLOMAX 20/20 (Dual-luciferase data); LSM510 (Bifc data); Tanon 5200 (Western Blot data).
Data analysis	Shim-pack UFLC SHI-MADZU CBM30A system; Shimadzu LC-16 system; Agilent UPLC1290-6540UHD Q-TOF; ZEN; TASSEL 5; SAS software version 9.4; MEGA software version 6.06.

For manuscripts utilizing custom algorithms or software that are central to the research but not yet described in published literature, software must be made available to editors and reviewers. We strongly encourage code deposition in a community repository (e.g. GitHub). See the Nature Portfolio guidelines for submitting code & software for further information.

Data

Policy information about availability of data

All manuscripts must include a data availability statement. This statement should provide the following information, where applicable:

- Accession codes, unique identifiers, or web links for publicly available datasets
- A description of any restrictions on data availability
- For clinical datasets or third party data, please ensure that the statement adheres to our policy

The amino acid sequences of UGTs were downloaded from NCBI databases and are available at the following URL: https://www.ncbi.nlm.nih.gov/. The GWAS

genotype data are available on Rice Diversity Project (http://www.ricediversity.org/data/index.cfm). All data supporting the findings of this study are available within the paper and its Supplementary Information. Source data are provided with this paper.

Human research participants

Policy information about studies involving human research participants and Sex and Gender in Research.

Reporting on sex and gender	Not applicable.
Population characteristics	Not applicable.
Recruitment	Not applicable.
Ethics oversight	Not applicable.

Note that full information on the approval of the study protocol must also be provided in the manuscript.

Field-specific reporting

Please select the one below that is the best fit for your research. If you are not sure, read the appropriate sections before making your selection.

☒ Life sciences ☐ Behavioural & social sciences ☐ Ecological, evolutionary & environmental sciences

For a reference copy of the document with all sections, see nature.com/documents/nr-reporting-summary-flat.pdf

Life sciences study design

All studies must disclose on these points even when the disclosure is negative.

Sample size	Sample sizes were determined based on previous reports and best practices.
Data exclusions	No data were excluded from the analyses.
Replication	All experiments were performed with at least three independently replicates . All attempts at replication were successful.
Randomization	All samples were arranged randomly into experimental groups.
Blinding	Due to the nature of the experimental setup blinding was not practical.

Reporting for specific materials, systems and methods

We require information from authors about some types of materials, experimental systems and methods used in many studies. Here, indicate whether each material, system or method listed is relevant to your study. If you are not sure if a list item applies to your research, read the appropriate section before selecting a response.

Materials & experimental systems

n/a	Involved in the study	
☐	☒	Antibodies
☒	☐	Eukaryotic cell lines
☒	☐	Palaeontology and archaeology
☒	☐	Animals and other organisms
☒	☐	Clinical data
☒	☐	Dual use research of concern

Methods

n/a	Involved in the study	
☒	☐	ChIP-seq
☒	☐	Flow cytometry
☒	☐	MRI-based neuroimaging

Antibodies

Antibodies used	The antibodies were provided by Cell Signaling Technology, GST-Tag (26H1) Mouse mAb#2624S, Lot: 7; MBP Tag (8G1) Mouse mAb #2396S, Lot: 6.
Validation	The Anti-GST, details, https://www.cellsignal.cn/products/primary-antibodies/gst-tag-26h1-mouse-mab/2624?site-search-type=Products&N=4294956287&Ntt=2624s&fromPage=plp&_requestid=376505The Anti-MBP, details, https://www.cellsignal.cn/

products/primary-antibodies/mbp-tag-8g1-mouse-mab/2396?_=1680935550536&Ntt=2396s&tahead=true. Atibodies are commercially available from the manufacturers.
